# Evaluation of LAMP for the diagnosis of *Loa loa* infection in dried blood spots compared to PCR-based assays and microscopy

**DOI:** 10.1590/0074-02760210210

**Published:** 2022-02-11

**Authors:** Thuy-Huong Ta-Tang, Pedro Berzosa, José Miguel Rubio, María Romay-Barja, Policarpo Ncogo, Diego Agudo, Zaida Herrador, Laura Cerrada-Gálvez, Agustín Benito

**Affiliations:** 1 National Centre of Tropical Medicine, Institute of Health Carlos III, Malaria and Neglected Tropical Diseases Laboratory, Madrid, Spain National Centre of Tropical Medicine Institute of Health Carlos III Malaria and Neglected Tropical Diseases Laboratory Madrid Spain; 2 National Microbiology Centre, Institute of Health Carlos III, Malaria and Emerging Parasitic Diseases Laboratory, Madrid, Spain National Microbiology Centre Institute of Health Carlos III Malaria and Emerging Parasitic Diseases Laboratory Madrid Spain; 3 Ministry of Health, Malabo, Equatorial Guinea Ministry of Health Malabo Equatorial Guinea; 4 Fundación Estatal, Salud, Infancia y Bienestar Social, Madrid, Spain Fundación Estatal Salud, Infancia y Bienestar Social Madrid Spain; 5 Universidad Complutense de Madrid, Facultad de Ciencias Biológicas, Madrid, Spain Universidad Complutense de Madrid Universidad Complutense de Madrid Facultad de Ciencias Biológicas Madrid Spain; 6 Cooperative Research Network on Tropical Diseases (Red de Investigación Colaborativa en Enfermedades Tropicales), Madrid, Spain Cooperative Research Network on Tropical Diseases Red de Investigación Colaborativa en Enfermedades Tropicales Madrid Spain

**Keywords:** filariae, Loa loa, DBS, molecular diagnosis, LAMP, real-time PCR

## Abstract

**BACKGROUND:**

*Loa loa* is a filarial species found exclusively in West and Central Africa. Microscopy is the traditional diagnosis method for human loiasis. Several molecular methods have developed as an alternative approach for identification of *L. loa* filarial parasites.

**OBJECTIVES:**

The aim of this study was to evaluate a Loa-Loop-mediated isothermal amplification (LAMP) assay to diagnose loiasis disease on dried blood spots (DBS) samples, compared to microscopy, filaria-real time-polymerase chain reaction (PCR) and nested-Loa PCR.

**METHODS:**

A total of 100 DBS samples and 100 blood smears were used for this study. DNA was extracted using saponin/Chelex method. DNA isolated was assayed by a Loa-LAMP assay in parallel to microscopy, filaria-real time PCR and nested-Loa PCR. The sensitivities and specificities of Loa-LAMP assay was computed comparing to each one of the reference methods.

**FINDINGS:**

Loa-LAMP’s sensitivity was more than 90% and specificity was nearly 100% when compared to molecular methods. On the other hand, sensitivity was decreased a bit when Loa-LAMP faced microscopy, but keeping the other statistical values high.

**MAIN CONCLUSIONS:**

Loa-LAMP is an appropriate method for loiasis diagnosis in endemic areas. Though, it has disadvantages like the reagents’ high price at the moment and not to be able to detect more filarial species at once.

Human filariasis is a group of diseases produced by parasitic worms called filariae. These parasites are nematodes (roundworms) and are found in different adult tissues, depending on the species.^1^^,^^2^^,^^3^^,^^4^^,^^5^ Filarial infections are some of most prevalent infections in the tropics, and two of them, namely onchocerciasis and lymphatic filariasis (LF), are classified amongst the neglected tropical diseases (NTDs) by the World Health Organization (WHO).^6^^,^^7^

*Loa loa* is a human filarial species, found exclusively in Western and Central Africa and transmitted by the bite of the *Chrysops* deerfly. This filarial worm causes loiasis, also known as African eye worm.^8^ In several endemic areas, *L. loa* co-exists with *Onchocerca volvulus*, *Wuchereria bancrofti* and *Mansonella perstans*.^5^^,^^7^^,^^9^^,^^10^

Although *L. loa* was traditionally an absent filaria for the WHO, it began to receive increasing attention when severe adverse events presenting as encephalopathy occurred in onchocerciasis control projects supported by the African Programme for Onchocerciasis Control following treatment with ivermectin. Hence, in areas where loiasis is co-endemic with onchocerciasis and/or LF, the ability to safely carry out mass drug administration with ivermectin is impeded, especially at very high microfilarial loads (˃ 30,000 microfilariae per millilitre (mf/mL)).^8^^,^^11^ Despite the important role of *L. loa* in onchocerciasis and/or LF co-infections, this filarial parasite is not included in the WHO’s list of NTDs. However, the Expanded Special Project for Elimination of Neglected Tropical Diseases (ESPEN), established in May 2016, which is a project dedicated to the control and elimination of the five NTDs amenable to preventive chemotherapy (LF, onchocerciasis, schistosomiasis, soil-transmitted helminthiasis and trachoma) does include *L. loa* in the list of diseases to be eliminated.^12^

Conventionally, laboratory diagnosis of human loiasis relies on the detection of mf on Giemsa-stained or haematoxylin/eosin-stained thick and/or thin blood films by microscopy, with or without prior concentration.^13^ Based on morphological descriptions, *L. loa* mf are long and thick (205-300 x 6-8 µm),with a sheath and a terminal nucleus in their tail.^2^^,^^4^^,^^13^^,^^14^^,^^15^ However, microscopy has major disadvantages, as it is a time-consuming, labor intensive and tedious method. The efficacy of mf detection is further decreased by the long pre-patency and the possibility of mild or occult loiasis, in which the load of mf parasites is undetectable microscopically.^1^^,^^4^

Several polymerase chain reaction (PCR) techniques have been developed to detect Loa-filarial infection in humans, with a high accuracy for detecting single or mixed infections with other filarias. As such, they have been shown to be an excellent alternative diagnostic method to microscopy. However, PCR methods have the disadvantage of requiring expensive equipment, laboratories with good infrastructure, a reliable electrical supply and highly trained staff, in addition to a long amplification process.^16^^,^^17^^,^^18^^,^^19^

Loop-mediated isothermal amplification (LAMP) for *L. loa* pathogen has also been developed to overcome the limitations of the PCR techniques. This molecular method is relatively recent, having emerged in 2000^20^ as a field-friendly and cost-effective diagnostic tool that requires no major capital equipment, naked eye detection of amplification by turbidity, colorimetry or fluorescence with the use of an intercalating DNA dye.^21^^,^^22^ Like real-time PCR, LAMP is a one-step amplification reaction but with the advantage of amplification under isothermal conditions.^23^

As such, the LAMP assay has emerged as a new molecular method with the potential to replace PCR methods in the near future given its numerous advantages and outstanding efficiency.^23^^,^^24^ However, its usefulness must be tested in each case since it may also have limitations that should be analysed, including the origin and conservation of the samples.

This study has been performed as the National Centre of Tropical Medicine (Madrid, Spain) carries out international scientific-technical cooperation programs with African countries where this disease is found. Recently, the Centre was awarded several projects to assess human filariasis transmission in Equatorial Guinea and develop national recommendations to move towards onchocerciasis elimination and LF/loiasis control at a country level.

The aim of this study was to evaluate the usefulness of a previously described LAMP assay to diagnose loiasis disease in stored samples, preserved as dried blood spot (DBS), compared to the reference methods used in the laboratory for the diagnosis of loiasis disease, namely microscopy, filaria-real time-PCR and nested-loa PCR.

## MATERIALS AND METHODS

*Clinical samples* - DBS and thick and thin blood smear (on the same slide) samples were obtained from the Laboratory of the National Centre of Tropical Medicine’s repository (collection number C.0005278/ISCIII/Spain), which is registered according to the Spanish Law RD 1716/2011 (article 22. 1). Both DBS and thick and thin blood smears belonged to the malaria project PREVAMAL (2013), to provide baseline data on malaria prevalence in Equatorial Guinea.^25^ Blood samples for each participant were taken from the finger and spotted onto Whatman 903™ paper (GE Healthcare Bio-Sciences Corp.) for molecular diagnosis and onto a clean, grease-free microscope slide for microscopy diagnosis. DBS samples had been stored in double zip-lock plastic bags with silica gel absorbent at -20ºC. Thick and thin blood smears had been stained with 10% Giemsa solution for 10 min. Although there were more than 1700 DBS and stained thick and thin blood smears in this malaria project, only 100 paired samples were selected for this study (100 DBS samples plus their corresponding thick and thin blood smears), using an adequate preservation and very good quality of the slides, and availability of a sufficient quantity of blood on the DBS to be extracted, as the main selection criteria.

*Microscopy* - The thick and thin blood smears were used to characterise the mf and calculate the microfilaraemia taking into account that each thick and thin blood smear contained approximately 20 and 5 µL, respectively. Morphological identification for mf was performed in line with published guidelines^14^ and examined by expert microscopists from the Laboratory of the National Centre of Tropical Medicine. Microfilariae densities were expressed as microfilariae per milliliter of blood (mf/mL) under a 10x magnification, and the filarial species was determined at a 100x magnification with immersion oil. All fields were examined before declaring a slide negative. The microfilariae densities quoted are the average value for the thick and thin films by microscopic examination.

*DBS* - Positive and negative controls were prepared using a fresh whole blood sample in EDTA, supplied by the Malaria and Emerging Parasitic Diseases Laboratory (Madrid, Spain). Thus 70 µL of blood was spotted onto Whatman filter paper and the spot allowed to dry in air for one day. DBS from infected and uninfected persons was previously diagnosed by microscopy on a blood smear. For each set of extractions performed, positive and negative DBS samples, as well as a blank (filter paper without blood), were always executed in parallel with the undiagnosed samples.

*DNA extraction* - DNA was extracted from DBS using the classical saponin/Chelex method, with slight modifications from the original protocol.^26^ Two filter paper discs measuring 5 mm in diameter were punched from the center of a circle using handheld hole puncher. These discs were immediately placed into a 1.5 mL-tube, 1 mL of 0.5% saponin (Fluka Biochemika. Sigma-Aldrich Chemie GmbH) in autoclaved phosphate-buffered saline (PBS) 1x was added, the resulting solution mixed thoroughly 2-3 times and incubated overnight at 4ºC or at 37ºC for 1 h. The brown solution obtained was aspirated and replaced with 1 mL of autoclaved PBS 1x; the tube was then incubated for 30 min at 4ºC. During this step, 100 mL of a solution of 5% Chelex-l00 (Bio Rad, Richmond, CA) in water was heated at 100ºC in a magnetic stirrer. After aspiration of the PBS 1x, 200 µL of hot Chelex was added to the two paper discs, vortexed at high speed for 30 s, and placed in the heatblock at 98ºC for 10 min with a brief vortex again, once during and once after the incubation. After centrifugation at full speed (13,000 rpm) for 2 min, the supernatant was carefully recovered so as not to remove any Chelex. The isolated DNA was centrifuged again at full speed under the same conditions to remove any remaining Chelex and collected into a new tube. Supernatants were used for any DNA-based technique immediately, stored at 4ºC for use in the following few days or at -20°C for a long future analysis.

Filter paper samples were handled according to safety procedures to prevent contamination of samples with each other by disinfecting the handle punch in NaOH 5 M solution and rinsing the residual NaOH with distilled water.

Prior to molecular testing, the isolated DNA samples should be centrifuged at 13,000 rpm for 2 min to place the remaining Chelex at the bottom of the tube. Chelex-100 is a chelating resin with a high affinity for multivalent metal ions, so it can inhibit the PCR by binding to Mg^2+^ ions.^27^

The quality of the DNA obtained using the saponin/Chelex extraction method was checked using Nested-Malaria PCR method, which uses the human small subunit ribosomal RNA gene sequences as internal control.^28^^,^^29^

*Molecular methods* - The presence of *L. loa* parasites in the DNA isolated was subsequently assayed using three different molecular methods, targeting different genes to diagnose loiasis infections, as indicated briefly below:

- Filaria-real time-PCR (F-RT-PCR): targeting the internal transcribed spacer one (ITS1) of the nuclear ribosomal gene of all filarial species.^17^ In this study, the annealing temperature was varied by 48ºC and the PCR mixture used was Luna^®^ Universal qPCR Master Mix 2x (New England Biolabs). The cycling conditions comprised an initial denaturation step at 95ºC for 1 min, followed by 45 cycles of denaturation at 95ºC for 15 s, annealing at 48ºC for 20 s and extension at 60ºC for 30 s. A sample was considered positive for filarial parasite if the melting temperature (Tm) curve of the amplified fragments was Tm =77ºC ± 0.5ºC and the species identification was according to amplified product size after analysing in a multicapillary electrophoresis.

- Nested-Loa PCR: this nested PCR targets the gene encoding the *L. loa* 15-kD protein.^18^ The original protocol was followed, except for the volume of DNA template: 5 µL was used for the first PCR round and 2 µL of the first amplification product was used for the nested PCR.

- Loa-LAMP: the isothermal amplification assay was designed to target the *L. loa*-specific gene sequences LLMF72.^22^ The LAMP reaction was performed using a WarmStart Colorimetric LAMP 2x Master Mix (New England Biolabs) DNA amplification kit in a total volume of 25 µL (20 µL of reaction mixture and 5 µL of DNA template). Primer concentrations were the same as calculated in the original protocol without modifications. All reactions were performed at 65ºC for up to 30 min and heated at 80ºC for 5 min, inactivating the *Bst* DNA polymerase. Positive amplification results were detected by the naked eye based on the colour change using the pH-sensitive dye Phenol red.

For each reaction performed, appropriate positive and negative controls were always included. Positive controls were DBS samples of *L. loa-*microscopy positive individuals; negative controls were DBS samples of *L. loa*-microscopy negative individuals from filarial-endemic regions who were negative for filarial infection but could be positive or negative for other tropical parasites, such as *Plasmodium* spp.

All samples were run in duplicate. If the duplicates varied (one positive, one negative), the samples were rerun in triplicate.

All F-RT-PCR reactions were performed using a Rotor Gene Q 5plex (QIAGEN GMBH, Germany). The remaining DNA amplifications, including the Loa-LAMP, were performed using an Applied Biosystems GeneAmp^®^PCR System 2700. Amplification products were detected using QIAxcel Advanced (QIAGEN GMBH, Germany), an automated system for analysing DNA fragments based on multicapillary electrophoresis.

See Table I for a more detailed overview of primer sequences, target gene, product size and other characteristics of the assays.

**TABLE I:** Main characteristics of the three molecular methods used in this study and its corresponding publication

Molecular method	Primer name	Primer (µM)	Sequence (5’-3’)	Annealing Tª	Target	Product size (bp)	Specificity	Publication
F-RT-PCR	FIL2-F	0.375	GGTGAACCTGCGGAAGGATC	48°C	ITS-1	286-344*	Universal	(17)
	FIL 2-Loa	0.375	GGTGAACCTGCRGMWGGATC				*Loa loa*	
	FIL2-R	0.75	TGCTTATTAAGTCTACTTAA				Filaria	
Nested-Loa PCR	Forward 15r3_1_	1	AATCAGGCAAATAATGGCACAAAA	65°C	15-kD protein	396	*Loa loa*	(18)
	Reverse 15r3_2_	1	GCGTTTTCTTCTCACCAGCTGTCT					
	Forward 15r3_3_	1	GGCACAAAACACTGCAGCAGTCCT	65°C		366		
	Reverse 15r3_4_	1	CAGCTGTCTCAAATCGAAGATTCT					
Loa-LAMP	F3	0.2	AGATTTGACGGCAACGGAAG	65°C	LLMF72	pH-sensitive dye Phenol Red: color change from pink to yellow	*Loa loa*	(22)
	B3	0.2	GCGTCAGTTTCGTGTTGTGA					
	FIP	2	CCGGAATCAGAGGAACGCTTGATCAACGTCAGAAATCAGCCA					
	BIP	2	GCACAGCAGAGTCTTCTAGTGGCGTTGATGACGCTCCCAA					
	LF	1	GGTGATGTAAAAGCAGGCTGT					
	LB	1	TAAGTTTTCCAGGAACTGCACC					

*: size depending filarial species (bp): Onchocerca volvulus 344; B. malayi 324; Mansonella perstans 312; Mansonella ozzardi 305; Wuchereria bancrofti 301; Loa loa 286.

*DNA sequencing and sequence analysis* - All the products amplified by F-RT-PCR and Nested-Loa PCR techniques were purified using Speedtools PCR Clean Up Kit 250 rxns (Biotools, B&M Lab, SA), then sequenced in both directions using Big Dye Terminator v3.1 Cycle Sequencing in an ABI PRISM^®^ 3700 DNA Analyzer. Sequences were compared to the genbank database using basic local alignment search tool (BLAST)^30^ and aligned using CLUSTAL W^31^ to confirm the diagnosis and detect possible mutations.

In the case of the Loa-LAMP method, the positive post-LAMP reaction mixture subjected to gel electrophoresis produces many bands of different sizes in a reproducible ladder-like pattern and these products could not be sequenced.

*Statistical analysis* - Statistical values [sensitivity, specificity, positive predictive value (PPV), negative predictive value (NPV) and kappa index] for the Loa-LAMP assay was calculated compared to each of the reference methods using the free software WinEpi: Working in Epidemiology.^32^ The confidence intervals (CI) were established at 95%.

## RESULTS

Overall, 66 samples were identified as negative by all the tests and 34 samples were positive in at least one of the methods tested (microscopy or molecular test). A list of positive samples has been created to facilitate the following and understanding of the results (Table II).

**TABLE II:** List of positive samples with their corresponding results obtained by microscopy and molecular methods

Samples	Microscopy	Microfilaremia (mf/mL)	F-RT-PCR	NESTED-LOA PCR	LOA-LAMP
34	L	1100	L	L	L
57	L	300	L	L	L
65	L	500	L	L	L
90	L	2200	L	L	L
98	L	3600	L	L	L
144	L	12200	L	L	L
169	L	400	N	N	N
179	L	500	L	N	N
220	L	2000	L	L	L
254	L	11600	L	L	L
297	L	450	N	N	N
301	L	5600	L	L	L
319	L	1900	L	L	L
79	Mp	200	N	N	N
133	Mp	600	Mp	N	N
149	Mp	800	Mp	N	N
164	Mp	100	Mp	N	N
176	Mp	100	Mp	N	N
192	Mp	1300	Mp	N	N
306	Mp	3200	Mp	N	N
308	Mp	100	Mp	N	N
310	Mp	400	Mp	N	N
326	Mp	1000	Mp	N	N
341	Mp	1000	Mp	N	N
194	L+Mp	200;200	N	N	N
276	L+Mp	200;200	L+Mp	L	L
318	L+Mp	6000;1500	L+Mp	L	L
74	N	0	Mp	N	N
76	N	0	Mp	N	N
104	N	0	Mp	N	N
106	N	0	Mp	N	N
137	N	0	Mp	N	L
138	N	0	Mp	N	N
141	N	0	Mp	N	N

F-RT-PCR: Filaria-real time-polymerase chain reaction; L: *Loa loa*; Mp: *Mansonella perstans*; N: negative. For a better visualisation of the results, each type of infection has a different padding. Microfilaremia in mixed infections: the first value is for *L. loa*, the second value is for *M. perstans*.

*Microscopy* - Out of 100 thick and thin blood smears analysed by microscopy, 73 (73%) were negative and 27 (27%) were positive: 13 were *L. loa*, 11 *M. perstans* and 3 were mixed infections (*L. loa* + *M. perstans)*. The average microfilaremia for *L. loa* was 3047 mf/mL (parasite count range 200-12200 mf/mL) and for *M. perstans* was 764 mf/mL (parasite count range 100-3200 mf/mL) (Table II).

*F-RT-PCR* - This method was able to detect 30 (30%) positive samples: 11 *L. loa*, 17 *M. perstans* and two mixed infections. Seven *M. perstans*-F-RT-PCR positive samples were missed by microscopy, whereas four microscopy positive samples were missed by F-RT-PCR (2 *L. loa*, 1 *M. perstans* and 1 mixed infection) (Table II).

*Nested-Loa PCR* - This method detected 12 (12%) *L. loa* positives and 88 (88%) *L. loa* negatives (Table II). Four samples *L. loa*-microscopy positives (3 *L. loa* single and 1 mixed infection) were missed by Nested-Loa PCR.

*Loa-LAMP* - This method detected 13 (13%) *L. loa* positives and 87 (87%) *L. loa* negatives, and missed the same four samples as Nested-Loa PCR (Table II). A sample was considered positive for *L. loa* if the color changed from pink to yellow (Figure).

**Figure:**
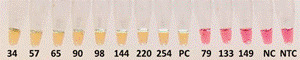
Loa-LAMP assay: DBS samples with their corresponding number assigned in the laboratory. The WarmStart Colorimetric LAMP 2x Master Mixes contains the pH-sensitive dye Phenol Red that changes color from bright pink (negative amplification for *L. loa*) to yellow (positive amplification for *L. loa*) as shown here after amplification for 30 minutes of. PC: positive control (positive for *L. loa* by microscopy and F-RT-PCR). NC: negative control (negative for any filarial parasite); NTC: non-template control.

*Statistical analysis* - Loa-LAMP’s sensitivity was more than 90% and the specificity was nearly 100% when compared to molecular methods (F-RT-PCR and Nested-Loa PCR). The concordance (kappa index) was good or excellent. On the other hand, sensitivity (75%) was slightly lower when comparing Loa-LAMP with microscopy, although the other statistical values remained high (Table III).

**TABLE III:** Statistical values obtained for Loa-LAMP assay comparing to microscopy, Filaria-real time-polymerase chain reaction (F-RT-PCR) and Nested-Loa PCR, references methods used in the laboratory

		Microscopy	F-RT-PCR	Nested-Loa PCR
Loa-LAMP	Sensitivity % (95% CI)	75.0% (53.8%, 96.2%)	92.3% (77.8%, 106.8%)	100.0% (100.0%, 100.0%)
	Specificity % (95% CI)	98.8% (96.5%, 101.1%)	98.9% (96.6%, 101.1%)	98.9% (96.6%, 101.1%)
	PPV % (95% CI)	92.3% (77.8%, 106.8%)	92.3% (77.8%, 106.8%)	92.3% (77.8%, 106.8%)
	NPV % (95% CI)	95.4% (91.0%, 99.8%)	98.9% (96.6%, 101.1%)	100.0% (100.0%, 100.0%)
	Kappa index % (95% CI)	79.9% (62.7%, 97.1%) *	91.2% (79.0%, 103.3%) **	95.4% (86.5%, 104.3%) **

PPV: positive predictive value; NPV: negative predictive value; CI: confidence intervals; *: good agreement; **: excellent agreement.

## DISCUSSION

The *L. loa* parasite is no longer as insignificant as it used to be in the past and its growing importance is due to the marked negative effect caused in people who live in co-endemic areas with *O. volvulus* and *W. bancrofti*, where mass drug administration is currently underway.^8^^,^^18^^,^^22^ As the goal of ESPEN is to accelerate elimination of the five most prevalent NTDs (LF, onchocerciasis, soil-transmitted helminthiases, schistosomiasis and trachoma) accurate *L. loa* parasite detection is essential for addressing the problem where *O. volvulus*, *W. bancrofti* and *L. loa* infections are co-endemic to achieve global elimination goals for *O. volvulus* and *W. bancrofti*.^7^^,^^12^

Microscopy on stained thick and/or thin blood smears remains the gold standard for routine clinical diagnosis of human loiasis in remote areas, due to the low cost of the materials, the ease with which it is performed, the minimal infrastructure required, and the ability to quantify the microfilaremia. However, this approach has limited sensitivity and specificity, and is not suitable for large-scale mf screening in disease-endemic areas.^33^^,^^34^

In the few last decades, several molecular methods, such as real-time PCR, Nested-PCR and LAMP, have been reported for the diagnosis of human loiasis infection with high sensitivity and specificity.^16^^,^^17^^,^^18^^,^^22^ These methods can easily diagnose infection by *L. loa* even if the parasite cannot be detected microscopically.

In this study, the utility of a Loa-LAMP method, developed by Drame et al. in 2014,^22^ was evaluated and compared to microscopy and two validated and well-known molecular methods, namely F-RT-PCR^17^ and Nested-Loa PCR,^18^ on 100 DBS. As F-RT-PCR and Nested-Loa PCR are not convenient methods for *L. loa* pathogen detection in the field and at a point-of-care setting, a Loa-LAMP assay was evaluated in light of the very promising results obtained by Drame et al.^22^

Loa-LAMP and the two other PCR assays described previously were developed for different targets of the parasite, and each one is probably represented by different numbers of copies in the filarial genome. These three selected methods targeting different genes might be an advantage, since if one clinical sample is positive for *L. loa* by all three methods, this confirms that the patient had loiasis infection.

A good diagnostic test should have a high validity, expressed as a function of sensitivity and specificity, which means that the percentages of false-positive and false-negative results should be limited, and that the different targets would have no influence on the statistical values. To calculate the relative sensitivity and specificity of the Loa-LAMP test, microscopy, F-RT-PCR and Nested-Loa PCR were taken as the standard test, with an estimated validity of approximately 100%.

The samples that were *L. loa*-microscopy single-positive but were missed by molecular methods may have had a very low mf load (≤ 500 mf/mL). Blood samples with very low microfilaremia have a lower possibility of taking one microfilariae in the punch because the distribution of mf on the blood spot is not homogenous. Thus, parasite material seems to be less concentrated towards the extreme edges of the blood spot, as shown in the experiment carried out by Baidjoe et al.^35^ Among the three mixed infections (*L. loa* + *M. perstans*) detected by microscopy, no loa parasite could be detected in one sample by any of the three molecular methods. This mixed infection also had a very low microfilaremia (200 mf/mL).

False-negative results due to the presence of potential enzyme inhibitors in the samples, or the poor DNA quality or concentration yielded by the saponin/Chelex DNA extraction method, were discarded, since all of them were checked with an internal extraction control present in the Nested-Malaria PCR method.^28^^,^^29^

Samples infected by *M. perstans* were also included in this study to identify possible cross-reactivity with *M. perstans* using the Loa-LAMP. There was only one case (#137) that tested *M. perstans*-F-RT-PCR positive and positive for *L. loa* by Loa-LAMP. This could be a possible mixed infection in which each method was able to detect the filarial parasite for which it is most sensitive. Overall, Loa-LAMP did not exhibit a cross-reaction with *M. perstans* DNA, although the possibility of a cross-reaction cannot be excluded.

Only one *M. perstans*-microscopy positive (#79) was missed by F-RT-PCR, probably because this sample had 200 mf/mL. Again, the sensitivity of the molecular method appeared to be compromised because of the low microfilaremia and the lower probability of collecting the microfilariae. It should be noted that F-RT-PCR detected seven more *M. perstans* which were not detected by microscopy.

After evaluating the Loa-LAMP assay, it has been found that the sensitivity and specificity are very high and the concordance between methods shows excellent agreement according to the kappa index values, thus suggesting that the method may be suitable for use with clinical samples in resource-limited areas endemic for loiasis.

As can be seen by the results, the Loa-LAMP assay may also be very useful as a potential point-of-care tool for the rapid amplification and easy detection of *L. loa* DNA in the field. Indeed, its statistical values are very similar to those found for F-RT-PCR and Nested-Loa PCR when tested with the same clinical samples.

Despite using a different source of samples, another method for DNA isolation and different reagents, the results obtained from the colorimetric LAMP assays in this study are similar to those reported by Drame et al.,^22^ with a high positive-predictive 92.3% (95% CI 77.8%-106.8%) and high negative-predictive [range 95.4% (95% CI 91%-99.8%)-100.0% (100.0%-100.0%), depending on the method used for comparison].

In our opinion, the major limitation of the LAMP assay is the price of the kit used. Thus, in our study, the cost per sample was 3.5-times higher than for real-time PCR and almost five-times higher than for Nested-Loa PCR. As such, this could be a major limitation for its use in developing countries. In addition, the Loa-LAMP method still needs electrical power for the water bath or heating block to carry out the reaction, and a freezer is needed to store the Loa-LAMP reagents, and not all the endemic areas have a continuous power supply.

Another disadvantage of this Loa-LAMP technique is that it is monoplex and, therefore, has a limited capacity to detect a wide variety of filarial species at once. Indeed, it has been specifically designed to detect *L. loa* DNA even though an absence of *L. loa* DNA does not rule out the presence of other blood microfilariae (*M. perstans* or *W. bancrofti*).

*In conclusion* - As illustrated by the results from this study, Loa-LAMP is very similar in terms of sensitivity and specificity to established diagnostic methods for detecting loiasis infection, such as microscopy, F-RT-PCR and Nested-Loa PCR. As such, it is an appropriate method for loiasis diagnosis if a rapid screening of patients in endemic areas is required. Moreover, Loa-LAMP is a good alternative for use in the field, as no major capital equipment is required, the turnaround times are short compared to F-RT-PCR and Nested-Loa PCR, and amplification by colorimetry can be detected by the naked eye, although the main disadvantages are the high price of reagents and the inability to detect more than one filarial species simultaneously. Given the good results for the diagnosis of LAMP, its applicability in the field and the ease with which positives can be detected, laboratories such as ours should design a LAMP that can detect more than one species simultaneously in order to facilitate work in the field. The basis of good scientific/technical cooperation, one of the responsibilities of our Centre, is to be able to offer endemic areas fast and reliable diagnostic tools that facilitate diagnosis, so that patients can quickly receive effective treatment.

*Ethics approval and consent to participate* - All the clinical samples, DBS and blood smears, had the authorisation of the director of National Centre of Tropical Medicine to be used in this study with the unique purpose to compare the Loa-LAMP assay to microcopy, and two molecular techniques, filaria-real time-PCR and nested-loa PCR.

List of abbreviations

BLAST: Basic Local Alignment Search Tool; bp: basepairs; ºC: degrees Celsius; CI: confidence intervals; DBS: dried blood spots; DNA: deoxyribonucleic acid; EDTA: ethylene diamine tetra-acetic acid; ESPEN: Expanded Special Project for Elimination of Neglected Tropical Diseases; ITS1: internal transcribed spacer one; LAMP: Loop-mediated isothermal amplification; LF: lymphatic filariasis; F-RT-PCR: filaria-real time-PCR; mf: microfilariae; mL: milliliter; mm: millimeter; min: minutes; NPV: negative predictive value; NTDs: neglected tropical diseases; PBS: phosphate-buffered saline; PCR: polymerase chain reaction; PPV: positive predictive value; rDNA: ribosomal deoxyribonucleic acid; RNA: ribonucleic acid; rpm: revolutions per minute; WHO: World Health Organization; s: seconds; Tm: melting temperature; µL: microliter.
